# ABO and Rh Blood Group Distribution in Kayseri Province, Turkey

**DOI:** 10.5505/tjh.2012.26918

**Published:** 2012-03-05

**Authors:** Yasemin Altuner Torun, Leyla Gül Kaynar, Çigdem Karakükcü, Mehmet Yay, Fatih Kurnaz, Hasan Mutlu, Mustafa Çetin, Bülent Eser

**Affiliations:** 1 State Educational and Research Hospital, Transfusion Center, Kayseri, Turkey; 2 Erciyes University, School of Medicine, Department of Hematology, Kayseri, Turkey; 3 State Educational and Research Hospital, Department of Biochemistry, Kayseri, Turkey

## TO THE EDITOR

Today, there are more than 600 antigenic structuresamong the 30 defined blood groups [1]. The ABO systemwas first described by Landsteiner in 1900, and remainsthe most important in the management of tissue and bloodtransfusion. Indeed, the Rh system has the most importantantigen D structure and is the most widely used prior totransfusion [1,2].

It has been shown that the frequency of ABO bloodgroups differs between populations [1,3]. Determinationof the frequency of blood groups in a particular regionfacilitates timely provision of blood and blood products.There are many studies on the distribution of blood groupsin Turkey [4-10]; however, none have considered KayseriProvince, a densely populated city in central Anatolia. Assuch, the present study aimed to determine the frequencyof blood group types in Kayseri Province in order tofacilitate to reach the appropriate blood type .

Blood samples obtained from 86,797 individuals thatpresented to blood banks run by Erciyes University,School of Medicine, State Educational and ResearchHospital, between January 2008 and September 2010were retrospectively analyzed. ABO group types andRh specificity of the blood samples were determinedusing a DiaMed-ID Micro Typing System (DiaMed AG,Switzerland) via gel centrifugation. Among the participants,45,134 (52%) were female and 41,663 (48%) were male.In all, 88.2% (n = 76,580) of the blood samples were Rhpositiveand 11.8% (n = 10,217) were Rh negative. Thefrequency of A, O, B, and AB blood group types was 44%(n = 38,253), 33.3% (n = 28,904), 16.2% (n = 14,031),and 6.5% (n = 5609), respectively. The frequency of ABOand Rh blood groups is shown in Figure 1.

ABO blood groups are determined based on a locus onchromosome 9¾the Rh gene is on the first chromosome[1]. The Rh system is clinically as important as the ABOsystem because of its role in hemolytic diseases in newbornsand in transfusion incompatibility [1]. ABO and Rh bloodgroups usually exhibit ethnic and regional differences[1,3]. The present findings show that the frequency of A,B, O, and AB blood group types in Kayseri is in accordancewith that in the general Turkish population.

In total, 44% of the blood samples analyzed in thepresent study were blood group A, which is similar tothe frequency in other central Anatolian cities, includingKonya, Eskişehir, and Ankara [4,5,6]. The frequency ofthe O blood group in Turkey is reported to be between30.8% and 41.2%. In the present study the frequencyof the O blood group was 33.3%, which is the same asin the Turkish cities Denizli and Diyarbakır [7,8], andthe frequency of the B type blood group in the presentstudy was 16.2%, which is similar to that in Eskişehir and Denizli [4,7]. In Turkey the highest frequency of theB blood group is in Şanlıurfa (21.25%) and the lowestfrequency is in Malatya (11.4%) [9]. The AB blood grouphas a frequency in Turkey of 6%-9.2%, which is similar tothe 6.5% observed in the present study.

The frequency of Rh-negative individuals variesenormously between ethnic groups. For example, 17% ofBrits are Rh negative, versus 0.5% of Japanese;3 however,the frequency of Rh negative in Turkey is nearly 15%,which is similar to that in Asia and Europe [1,2]. In thepresent study the Rh positivity rate was 88.2%, whereasother studies conducted in Turkey reported that the Rhpositivity rate was 90.8% in Gaziantep, 89% in Malatya,89.1% in Diyarbakır, and 89.9% in Denizli [8-10].

In conclusion, the frequency of the ABO and Rh bloodgroups observed in Kayseri Province in the present studydiffers from that in other Turkish cities, but is similar tothat in the general Turkish population. This could be dueto the fact that Kayseri Province is located in the center ofTurkey. In recent years Kayseri has experienced the influxof a high number or migrants coming from other regionsof Turkey (especially the south and east) due an increase inthe number of industrial jobs. According to Turkish nationwidepopulation statistics (Türkiye İstatistik Kurumu)for 2008-2009, 28,831 people migrated to Kayseri¾amigration rate of 1.86%. It is critically important thatblood banks know the distribution of blood group typesin their region. We think that the present study’s findings,as those from studies conducted in other Turkish cities,will contribute to the Turkish blood type database and,most importantly, will facilitate easy access to appropriateblood types, as needed.
